# The effect of compression stockings on the complaints well-being and sleep quality of pregnant women with restless legs syndrome: a randomized controlled study

**DOI:** 10.1590/1806-9282.20240145

**Published:** 2024-08-16

**Authors:** Özlem Kaplan, Mürüvvet Başer, Mahmut Tuncay Özgün

**Affiliations:** 1 Erciyes University, Faculty of Medicine, Department of Obstetrics and Gynecology – Kayseri, Turkey.; 2 Erciyes University, Faculty of Health Sciences, Department of Obstetrics and Gynecology – Kayseri, Turkey.

**Keywords:** Compression stockings, Obstetric nursing, Restless legs syndrome, Sleep quality, Quality of life

## Abstract

**OBJECTIVE:**

The aim of this study was to determine the effect of compression stockings on complaints, well-being, and sleep quality in pregnant women with restless legs syndrome.

**METHODS:**

This randomized placebo-controlled study was conducted on 63 pregnant women (placebo group [PG]=31; experimental group [EG]=32) at the Perinatology Outpatient Clinic of a Health Research and Application Centre in Turkey. Pregnant women in the experimental group wore compression stockings when they got up in the morning for 3 weeks and took them off at bedtime. Placebo group women wore a placebo stocking. Data were collected using the restless legs syndrome Severity Rating Scale, the Pittsburgh Sleep Quality Index, the World Health Organization-5 Well-Being Index, and the Application Satisfaction Form on the 22nd day of the first interview. Statistical significance was accepted as p<0.05.

**RESULTS:**

Post-test mean scores of both the experimental group and placebo group in the restless legs syndrome Severity Rating Scale (post-test:;8.87±5.27, 12.19±5.60; pre-test:;21.28±5.63, 21.0±5.61; p<0.05), the Pittsburgh Sleep Quality Index (post-test:;5.34±3.28, 6.12±3.12; pre-test:;10.15±4.23, 9.61±4.59; p<0.05), and Well-Being Index (post-test:;18.06±4.59, 19.00±4.47; pre-test:;12.71±5.85, 15.09±5.62; p<0.05) showed recovery according to the pre-tests. However, the post-test restless legs syndrome Severity Rating Scale of the experimental group was lower than that of the placebo group (p<0.05). The effect of their application started in 3.93±1.74 days on average in the experimental group, while it started in 5.09±1.55 days in the placebo group (p<0.05).

**CONCLUSION:**

Both applications reduced the severity of restless legs syndrome in pregnant women and increased sleep quality and well-being. However, compression stockings were more effective in reducing restless legs syndrome severity. Nurses can use compression and placebo stockings in the care of pregnant women with restless legs syndrome.

**Clinical Trial Registration Number:**

NCT05795868.

## INTRODUCTION

Restless legs syndrome (RLS) is a chronic sensory-motor disorder that causes an irresistible urge to move and discomfort in the legs. Symptoms begin, especially during long-term inactivity, such as sleeping and resting^1^. The syndrome is seen twice as often in women^2^. In addition, RLS is more common in pregnant women compared to other women, and it is seen in 15.4–29.2% of pregnant women in Turkey^3,4^.

It has been stated that genetic factors, the brain dopamine system, and pregnancy-specific factors such as multiparity, hemoglobin, iron and folate deficiency, estrogen level, and nerve tension may be influential in the formation of the syndrome during pregnancy^1,4–6^.

Although RLS is a condition that can be seen in every trimester, its incidence and severity peak in the third trimester^7^. It states that as symptoms worsen, there may be significant distress in sleep, well-being, cognitive health, activities of daily living, and social areas of essential functioning^5–9^. It was also found that there was an increased rate of pre-eclampsia, difficult labor, cesarean section, and depression in women with RLS. Early treatment is therefore essential for a healthier pregnancy and fetus^5,8–10^.

Nonpharmacological treatments are primarily recommended for RLS. One recommended treatment is to use a pneumatic compression device, proven to work in hemodialysis patients^5^. RLS symptoms in pregnant women can be relieved by using compression stockings, which can have a similar effect at the same pressure. However, no studies have been found in the literature to report the effectiveness of compression stockings on RLS symptoms. In this context, the aim was to determine the effect of compression stockings on the complaints, well-being, and sleep quality of pregnant women with RLS.

## METHODS

### Study design

We conducted a randomized, placebo-controlled study (Clinical Trials: NCT05795868).

### Settings and samples

**Figure 1:**
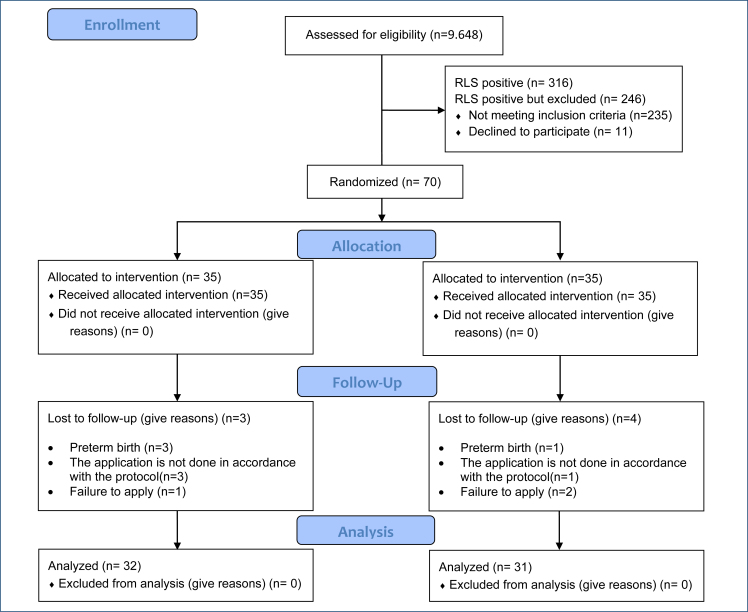
Consort flowchart.

This study was conducted in the perinatology outpatient clinic of a university hospital in Turkey. The sample size was determined to be 29 people in each group (differences=1.51; power=0.95; standard deviation=2.5; n2/n1=1)^11^. However, 35 pregnant women were included in each group to allow for data that might be lost. Notably, 70 women who met the inclusion criteria were allocated to groups according to pre-prepared randomization lists (fsl 1). Inclusion criteria for the study: the women should be RLS according to the RLS Diagnostic Criteria Questionnaire Form and doctor's examination, symptom severity >10, literate, ages of 18–40 years, with a single pregnancy at 27–37 weeks of gestation, take iron, vitamin D, magnesium, and calcium supplements, and have hemoglobin ≥11 g/dL. The women with communication barriers, high-risk pregnancy, pre-pregnancy RLS, chronic disease, body mass index (BMI) >30, sleep apnea, dermatological problem in the feet and legs, varicose veins, who used antipsychotic, antidepressant, or heparin, antihistamine, antiemetic, calcium channel blocker, dextromethorphan, and decongestant-type drugs were excluded from the study (Figure 1).

### Measures

Personal Information Form: The form consists of questions including socio-demographic and obstetric characteristics^2–7^. The RLS Diagnostic Criteria Questionnaire Form: The International Restless Legs Syndrome Study Group (IRLSSG) created the diagnosis form^12^. RLS is diagnosed by answering "yes" to all five questions^3,9^.

Restless legs syndrome Severity Rating Scale: The scale was developed by IRLSSG and consists of 10 questions^13^. The score range varies between 0 and 40. A score of 1–10 indicates mild, a score of 11–20 indicates moderate, a score of 21–30 indicates severe, and a score of 31–40 indicates very severe. The validity and reliability study of the scale was conducted in Turkey^14^. In this study, Cronbach's alpha coefficient was 0.79–0.90.

Pittsburgh Sleep Quality Index (PSQI): PSQI was developed in 1989 by Buyyse et al.^15^ It evaluates sleep quality. The PSQI consists of 24 questions. The total PSQI score ranges from 0 to 21. Scores greater than 5 indicate poor sleep quality. The validity and reliability study was conducted by Ağargün et al in Turkey^16^. In this study, Cronbach's alpha coefficient was found to be 0.76–0.79.

World Health Organization-5 Well-Being Index (WHO-5): The WHO-5 questionnaire comprises five items related to the participant's feelings. Each item is evaluated between 0 and 5. The total score ranges from 0 to 25. A total score below 13 indicates a poor quality of life^17^. The validity and reliability study of the scale was conducted in Turkey^18^. In this study, Cronbach's alpha coefficient was found to be 0.87–0.88.

Application Satisfaction Form: The researcher created the form to determine the participants’ satisfaction levels toward the application. Satisfaction levels consist of two parts: positive and negative feedback about the application is expressed numerically on a decimal scale and is open-ended.

### Procedures

The data collection instruments were administered to pregnant women in a face-to-face interview with the researcher. Data were collected in outpatient clinics and it took approximately 20–25 min to complete the questionnaires. The Personal Information Form, IRLSSG, PSQI, and WHO-5 forms were administered to all of the pregnant women as a pre-test. For 3 weeks, the women in the groups wore the socks they were given before. The women were administered the IRLSSG, PSQI, WHO-5, and a follow-up form at the end of 3 weeks.

#### Experimental group intervention

The experimental group (EG) used CCL2 (below-knee medium pressure) stockings (23–32 mm/Hg) with a graduated pressure system and size variation. The size of compression stockings suitable for pregnant women was determined, and stockings were provided. Women in groups were taught how to wear the stockings and were given written instructions. For 3 weeks, the women in the groups wore the socks they were given before getting up in the morning at home and took them off when they slept.

#### Placebo group intervention

The placebo group (PG) used 100-denier knee-high stockings with no therapeutic effect. Women in groups were taught how to wear the stockings and were given written instructions. For 3 weeks, the women in the groups wore the socks they were given before getting up in the morning at home and took them off when they slept.

### Statistical analyses

The data were analyzed using SPSS 24.0, and values p<0.05 were accepted. The normality of the data of the numerical variables was evaluated with the QQ plot, the kurtosis, and skewness measures. Homogeneity between groups was analyzed by t-test. Due to the normal distribution of the data, the independent sample t-test was used in the independent groups, and the dependent-sample t-test was used in the dependent groups. Descriptive analysis was used in cases that expressed feedback about the application.

### Ethical aspect of the study

Approval (2020/627) to conduct the study was obtained from the Clinical Research Ethics Committee, and the Helsinki Declaration ethical principles were followed at all stages. Informed voluntary written consent was obtained from those included in the study.

## RESULTS

The groups were similar in terms of socio-demographic and obstetric characteristics (p>0.05; Table 1).

**Table 1:** Comparison of socio-demographic and obstetric characteristics by groups.

Features	EG (n=32)	PG (n=31)	t p
	$\bar{\text{x}}$ ± SD	$\bar{\text{x}}$ ± SD	
Age (years)	27.75±4.99	26.48±5.54	0.953 0.344
BMI	26.93±2.65	26.65±3.03	0.393 0.696
Gestational weeks	30.78±3.82	30.12±3.67	0.690 0.493
Gravida	2.21±1.28	2.32±1.27	−0.321 0.749
Weight gained during pregnancy	9.03±3.64	7.77±3.33	0.833 0.159

t=independent sample test. EG: experimental group; PG: placebo group; SD: standard deviation; BMI: body mass index.

The IRLSSG pre-test mean scores of the EG and PG were similar (21.28±5.63 and 21.0±5.61, respectively; p>0.05). The post-test mean score of the EG (8.87±5.27) was lower than the PG (12.19±5.60) (p<0.05). However, the IRLSSG post-test mean score of both groups was statistically significantly lower than the pre-test mean scores of the groups (p<0.001; Table 2).

**Table 2:** Comparison of the pre-test and post-test mean scores of the scales according to the groups.

Scales		EG (n=32)	PG (n=31)	Test p
		$\bar{\text{x}}$ ± SD	x ± SD	
IRLSSG				
	Pre-test	21.28±5.63	21.0±5.61	0.198^t^ 0.843
	Post-test	8.87±5.27	12.19±5.60	−2.421^t^ 0.018
	Test p	11.625^y^ <0.001	7.285^y^ <0.001	
	Difference	12.40±6.03	8.80±6.73	0.381 0.029
PSQI total score				
	Pre-test	10.15±4.23	9.61±4.59	0.488^t^ 0.627
	Post-test	5.34±3.28	6.12±3.12	−0.971^t^ 0.336
	Test p	6.654^y^ <0.001	4.057^y^ <0.001	
	Difference	4.81±4.09	3.48±4.78	0.668 0.240
WHO-5 total score				
	Pre-test	12.71±5.85	15.09±5.62	−1.643^t^ 0.106
	Post-test	18.06±4.59	19.00±4.47	−0.876^t^ 0.105
	Test p	7.169^y^ <0.001	3.987^y^ <0.001	
	Difference	5.34±4.21	3.96±5.54	0.192 0.271
Benefit from the application				
	Yes	31 (96.9)	29 (93.5)	0.378x2 0.613
	No	1 (3.1)	2 (6.5)	
	Day of benefit *(x ± SD)	3.93±1.74	5.09±1.55	−2.784 0.007
	Satisfaction level (x ± SD)	8.18±1.61	8.16±1.61	0.064^t^ 0.949

* Responses were received from pregnant women who benefited from the application.

t Independent sample t-test.

y Paired sample t-test.

x2 Chi-square. EG: experimental group; PG: placebo group; IRLSSG: International Restless Legs Syndrome Study Group; PSQI: Pittsburgh Sleep Quality Index; WHO-5: World Health Organization-5 Well-Being Index; SD: standard deviation.

The PSQI pre-test scores of the groups were similar (p>0.05). Although the mean post-test PSQI score was smaller in the EG (5.34±3.28) than in the PG (6.12±3.12), this difference was not statistically significant (p>0.05; Table 2).

The groups were similar in terms of WHO-5 pre-test scores and post-test scores (p>0.05). The mean post-test WHO-5 score was similar in the EG (18.06±4.59) and PG (19.00±4.47) groups (p>0.05; Table 2). Women in the EG reported that they experienced relief, on average, 3.93±1.74 days after application, while women in the PG reported that they experienced relief, 5.09±1.55 days after application (p<0.05; Table 2).

The most common positive and negative codes in the EG were pain relief/reduction (n=13) and discomfort (n=14). For PG, the most commonly reported positive and negative codes were pain relief/reduction (n=15) and sweaty legs (n=4) (Table 3).

**Table 3:** Distribution of positive and negative statements of pregnant women in the experimental group and placebo group (n=32)*.

Codes		Participants																								
EG group positive statements		P-2	P-3	P-4	P-6	P-9	P-10	P-11	P-17	P-18	P-19	P-21	P-23	P-24	P-27	P-29	P-32	P-34	P-41	P-43	P-44	P-47	P-49	P-60	P-65	P-67
Reduction in leg pain, relief					x	x	x	x	x		X	x						x		x	x	x		x	x	
No pain in the legs			x	x						x			x	x		x			x				X			
Comfortable sleep			x	x		x	x		x		X	x	x	x	x			x	x							
Reduction in leg edema/low back pain																	x									
Psychological relief																					x					
EG group negative statements																										
	Sock slip	x				x		x																		
	Sock tightening		x	x	x				x	x	x	x		x			x	x	x							
	Difficulty wearing			x	x	x		x	x	x	x	x	x		x		x	x	x						x	
	Discomfort such as sweating and itching in the legs			x											x										x	
	Continuing leg pain							x																		x
**PG group positive statements**		**P-14**	**P-15**	**P-16**	**P-20**	**P-22**	**P-25**	**P-26**	**P-29**	**P-30**	**P-31**	**P-33**	**P-35**	**P-42**	**P-45**	**P-46**	**P-50**	**P-56**	**P-61**	**P-64**	**P-70**					
Reduction in leg pain, relief		x	x	x	x	x				x	x	x	x	x	x	x	x	x		x						
No pain in the legs							x	x	x																	
Comfortable sleep			x		x		x	x	x			x	x		x											
Getting one's life in order												x		x												
PG group negative statements																										
	Sock tightening															x					x					
	Difficulty wearing					x				x	x															
	Sweating in the legs					x						x		x		x										
	Continuing leg pain	x													x				x							

* One participant is listed under more than one code. P: participant.

## DISCUSSION

In the study, the severity of RLS decreased to a mild level in the EG after application, while it decreased to a moderate level in the PG. Although improvements in the severity of RLS were observed in both groups, this effect was greater in the EG. There is evidence that the compression device and enhanced external counterpulsation (EECP), which have a similar effect on the venous system, may be effective in groups other than pregnancy^11,19^. A study of 10 people found that using a pneumatic compression device for 1 h in the evening could relieve RLS symptoms and improve their quality of life^20^. In another randomized, placebo-controlled trial, people with RLS were given 1 h of therapeutic or placebo compression per day. The study found a beneficial effect of placebo compression. At the same time, therapeutic compression was found to improve the severity of the disease compared to placebo^11^. In their pilot study, Rajaram et al. ^19^ found that enhanced EECP for 35 days significantly improved RLS symptoms in six patients with RLS. These investigators then conducted a randomized, double-blind, and sham-controlled study of EECP in RLS patients. The study was completed with six people and found that although both groups experienced an improvement in RLS severity scores, this could not be statistically evaluated^21^. Based on the results of this study, it is suggested that pneumatic compression can be used to reduce the severity of RLS symptoms during pregnancy and that the placebo effect should be investigated^5^. In this context, the findings obtained in the study are similar to those found in the literature^19,20^. The fact that compression stockings reduce the severity of RLS supports the theory that RLS may be associated with venous insufficiency^19,20,22,23^. Pneumatic compression devices, compression stockings, and EECP are thought to reduce the sensory symptoms of RLS by affecting the peripheral or central nervous system by regulating vascular flow. The significant effect of the placebo socks is similar to that found in the literature and shows that psychological factors can affect the severity of RLS^11,20^.

The negative impact on sleep disturbance and quality of life in patients with RLS generally varies in direct proportion to the severity of the disease^6,8,9^. This study, like others, found that pregnant women in the groups experienced severe symptoms and had impaired sleep and quality of life. However, after the applications, the severity of RLS decreased in both groups, and there were similar improvements in sleep quality and quality of life. In a randomized, placebo-controlled study, positive effects of therapeutic or placebo compression applied to people with RLS were observed in both groups. However, in contrast to our study, therapeutic compression was found to improve daytime sleepiness, fatigue measures, and quality of life compared to placebo^17^. Another study evaluated polysomnographic measurements in six patients with RLS after EECP (four people) or placebo (two people) treatment and concluded that although there was a reduction in RLS severity, there was no significant effect on sleep^21^.

The reduction of RLS severity and symptoms during pregnancy is very important. However, characteristics such as time to start, ease of use, satisfaction, and positive or negative aspects of the effectiveness of the application should also be identified. This study reported that almost all pregnant women in both the intervention and placebo groups benefited from the application, with the effect of the application starting on average from day 4 in the intervention group and from day 5 in the placebo group. Satisfaction with the treatment was similar in both groups. Reduction/relief of leg pain was reported as a positive feature of the applications in both groups. However, there were more negative reports about the use of compression stockings. However, the reported negative effects were more related to discomfort due to the tightness of the stockings or the enlargement of the abdomen than to health problems.

### Strengths and limitations of the study

This research is one of the limited studies conducted to reduce the severity of symptoms in pregnant women with RLS. It is also the first study in the literature to investigate the effectiveness of compression stockings in reducing the severity of RLS. The results suggest that applying compression and placebo stockings may effectively reduce RLS symptoms and improve sleep and quality of life. In addition, how long the applications took effect and the women's opinions about the applications were also questioned. Another strength of this study is that compression and placebo stockings are cost-effective and easy to obtain. In addition, there is no need for hospitalization since technical knowledge and personnel are not needed for its use. Since people will not have to spend additional time or effort using socks, they can continue their daily activities. The limitation of the study is that the results were based on self-report rather than objective measurement.

## CONCLUSION

The study found that wearing compression stockings and placebo stockings reduced the severity of RLS in pregnant women and improved their sleep and quality of life. The effect of the stockings was felt within 3–5 days, and the women were satisfied with the stockings and did not report any serious complications. In this context, health professionals, especially nurses, should develop institutional policies to reduce the severity of symptoms in pregnant women with RLS and develop solutions, including the use of compression stockings.
